# An Overview of Pickering Emulsions: Solid-Particle Materials, Classification, Morphology, and Applications

**DOI:** 10.3389/fphar.2017.00287

**Published:** 2017-05-23

**Authors:** Yunqi Yang, Zhiwei Fang, Xuan Chen, Weiwang Zhang, Yangmei Xie, Yinghui Chen, Zhenguo Liu, Weien Yuan

**Affiliations:** ^1^Department of Neurology, Xin Hua Hospital Affiliated to Shanghai Jiao Tong University School of MedicineShanghai, China; ^2^School of Pharmacy, Shanghai Jiao Tong UniversityShanghai, China; ^3^Zhiyuan College, Shanghai Jiao Tong UniversityShanghai, China; ^4^Department of Neurology, Jinshan Hospital, Fudan UniversityShanghai, China

**Keywords:** Pickering emulsion, drug delivery, biomaterials, microspheres, nanoparticles

## Abstract

Pickering emulsion, a kind of emulsion stabilized only by solid particles locating at oil–water interface, has been discovered a century ago, while being extensively studied in recent decades. Substituting solid particles for traditional surfactants, Pickering emulsions are more stable against coalescence and can obtain many useful properties. Besides, they are more biocompatible when solid particles employed are relatively safe *in vivo*. Pickering emulsions can be applied in a wide range of fields, such as biomedicine, food, fine chemical synthesis, cosmetics, and so on, by properly tuning types and properties of solid emulsifiers. In this article, we give an overview of Pickering emulsions, focusing on some kinds of solid particles commonly serving as emulsifiers, three main types of products from Pickering emulsions, morphology of solid particles and as-prepared materials, as well as applications in different fields.

## Introduction

Emulsions are widely used in many different fields including pharmaceutics, drug delivery, cosmetics, food industry, and so on, especially after the advancement of methods for preparing various kinds of emulsions. It is commonly known that emulsions can be stabilized by small molecular emulsifiers and some macromolecules, yet some of them may cause allergy-like reactions and carcinogenicity. Pickering emulsion (Pickering, 1907) utilizes solid particles alone as stabilizers, which accumulate at the interface between two immiscible liquids (typically denoted as oil and water phase) and stabilize droplets against coalescence. It was discovered a century ago, but has recently drawn significant research interests as templates in many fields due to the following advantages: (i) solid particles reduce the possibility of coalescence, bringing about higher stability to emulsions; (ii) many solid particles can endow as-prepared materials useful characteristics such as conductivity, responsiveness, porosity, and so on; (iii) some food-grade solid particles have lower toxicity, thus leading to higher safety for *in vivo* usage. It has been demonstrated by many researches that numerous types of inorganic particles including silica, clay, and hydroxyapatite (Hap), as well as some organic particles, can effectively serve as Pickering emulsifiers.

Theories that demonstrate the mechanism of stabilization in Pickering emulsions have been proposed, and the commonly accepted one is based on the formation of a steric barrier by solid particles adsorbing at the oil–water interface (Monegier du Sorbier et al., 2015). That is, particles are able to irreversibly attach to the oil–water interface, leading to a more efficient stabilization than surfactant adsorption. This mechanism was supported by many scientists through theoretical approaches and some thermodynamic calculations (Menon and Wasan, 1988; Binks and Clint, 2002; Aveyard et al., 2003). Whether oil-in-water (O/W) Pickering emulsion or water-in-oil (W/O) Pickering emulsion can be formed is determined by the wettability of solid particles at the oil–water interface: if one of the liquids wets solid particles more than the other one, the better wetting liquid becomes the continuous phase and the other becomes the dispersed phase. O/W emulsions will come into being if the three-phase contact angle θ (angle at the three-phase boundary of solid particles, continuous phase and dispersed phase) is less than 90° (e.g., silica, clay), and W/O emulsions should form if θ > 90° (e.g., carbon black). However, only when θ is relatively close to 90° can the particle effectively act as a Pickering stabilizer. Since particles tend to remain dispersed in either phase if they are too hydrophilic (low θ) or too hydrophobic (high θ) (Binks and Lumsdon, 2000b). In this respect, many researches have been done to modify these particles with different molecules or to different degrees, in order to make them more amphiphilic.

In this review, various kinds of commonly used solid particles, including Hap, silica, clay, magnetic nanoparticles, chitosan (CS), cyclodextrin (CD), nanotube, and some food-grade stabilizers; types of materials fabricated from Pickering emulsions, including microsphere (MS), microcapsule (MC), and Janus colloidal particles (JCPs); morphology of solid particles being used and materials being fabricated, as well as applications of Pickering emulsions in delivery vehicles, porous scaffolds, stimuli-responsive materials, catalysts, and so on will be discussed in detail, with some recent researches.

## Solid Particles

The most notable difference between a Pickering emulsion and a classical emulsion is that, the former one bears solid particles on the interface between two liquid phases serving as the stabilizing agent, whereas the latter uses molecular surfactants to stabilize emulsions. So the stability, type (O/W or W/O), morphology, characters of Pickering emulsions are highly depended on the properties of solid particles. Therefore, it is significant to choose the right kind of nano/micro-particles, in order to obtain the specific type, character and application of Pickering emulsions. Several types of solid particles will be listed and discussed in detail.

### Hydroxyapatite

Hydroxyapatite [Ca_10_(PO_4_)_6_(OH)_2_] is an important component in human bodies, especially in bones and teeth, as the main mineral. Due to the excellent adsorbability with many compounds, Hap nanoparticles have already been extensively used in the formation of Pickering emulsions, which can be applied to a variety of applications such as biomaterials, adsorbents, and catalysts. Besides, Hap can be simply synthesized through different approaches, such as wet chemical process, sol-gel process, emulsion process, and so on (Fujii et al., 2007).

It was clarified by previous experiments that Hap nanoparticles could help form O/W Pickering emulsions when the oil contained an ester group or the oil phase contained other polymers with ester groups, whereas Hap nanoparticles alone could not work as an emulsifier for Pickering emulsions (Fujii et al., 2007, 2009). A latter study further confirmed that the interactions between end groups of polymers and Hap nanoparticles at the oil–water interface were crucial in stabilizing Pickering emulsions as well as regulating the size of droplets and the morphology of products; the researchers chose polystyrene (PS) molecules with diverse end groups (like carboxyl groups, ester groups) and molecular weights to investigate their influence on the formation of Hap nanoparticle-stabilized droplets and MSs (Okada et al., 2012). Another work also utilized the interaction between the Hap nanoparticles and carbonyl/carboxylic acid groups, while the creative part was to add poly(ε-caprolactone) (PCL) which could dissolve in a wider range of organic solvents compared with another two polyesters, poly(L-lactic acid) (PLLA) and poly(L-lactide-co-glycolide) (PLGA); this means that non-halogenated solvents can be used (Fujii et al., 2012).

Artemisia argyi oil (AAO) has wide applications because of its anti-bacteria and anti-inflammatory effect, which are partly blocked by its relatively instability confronting air, light, and heat. To solve this problem, Hu Y. et al. (2013) prepared AAO-loaded antibacterial MCs with Hap/poly(melamine formaldehyde) (PMF) composite as a Pickering emulsifier.

### Silica

Silica is one of the most extensively studied solid particles as Pickering emulsifiers because they are easily obtained and modified, especially regarding to the study of phase inversion of emulsions (Binks and Lumsdon, 2000a,c; Duan et al., 2009). Massive experiments indicated that unmodified silica tends to stabilize O/W Pickering emulsions due to the hydrophilicity resulting from Si-OH groups on particle surface, whereas hydrophobically modified silica preferentially stabilizes W/O Pickering emulsions (Binks and Lumsdon, 2000c). Therefore, many studies aimed to produce various kinds of modified silica, in order to get different properties for better application through Pickering emulsions, such as polymerization (Duan et al., 2009; Zhou H. et al., 2013).

Factors that influence silica-stabilized Pickering emulsions, such as pH and salt concentration, have been investigated systematically (Binks and Lumsdon, 1999). Given the fact that pure silica is too hydrophilic to stabilize Pickering emulsion at basic condition because of surface charge, and that particles are likely to aggregate at lower pH, proper molecules should be linked to bare silica so that stabilizing ability is improved while remaining modest surface charge. In one study, a fatty acid with certain biocompatibility, oleic acid, was chosen to solve the problem and led to relatively stable Pickering emulsions with different size range (Sadeghpour et al., 2013).

Besides, the use of SiO_2_ has another convenience, since the outer silica shell can get removed by simply washing with HF aqueous solution, so that bare polymer-MSs could be obtained with better biocompatibility (Wei et al., 2012a).

### Clay

Clay is one of the most popular candidate for the formation of Pickering emulsions, partly because unlike surfactants, they are non-pollutant, cheap, and easily obtained. In most previous researches, clays were pre-treated with organic or amphiphilic molecules, thanks to the siloxane surface, so as to promote the adsorptivity of clay particles on oil (Guillot et al., 2009).

Due to the hydrophilicity of clay-surface, it should be modified with some kinds of molecules in order to be able to stay at the interface between water and oil. In this respect, Reger et al. (2012) used laponite XLG with surfactants to form gel-like Pickering emulsion, the gel-pattern of which was explained from the bigger surface of clays, more amphiphilic molecules-covering, and stronger attraction between droplets.

Besides, one recent study revealed that laponite could strongly influence the polymerization of styrene through Pickering emulsion, where laponite platelets between two phases could not only guarantee system-stability, but predetermine the size and number density of products as well as the reaction rate; in addition, they carried on various kinds of analysis to find out that the clay platelets produced a thick shell around the PS particles by adsorbing as multilayers (Brunier et al., 2016).

### Magnetic Nanoparticles

In recent years, magnetic Fe_3_O_4_ nanoparticles have attracted great research attention, especially in biomedical field, due to the negligible toxicity and useful magnetic property. A great number of biomedical materials have been fabricated by means of Fe_3_O_4_ stabilized Pickering emulsions. The unmodified Fe_3_O_4_ nanoparticles are hydrophilic owing to the many hydroxyl groups on the particle surface, while they can be turned into hydrophobic through appropriate surface decoration.

There have been a number of studies using modified Fe_3_O_4_ to form Pickering systems, while for the first time, Zhou et al. (2011) investigated the results from unmodified Fe_3_O_4_ stabilizing two-phase system. Their experiments showed that hydrophilic Fe_3_O_4_ nanoparticles could only stabilize systems with non-polar or weakly polar oils, where the contact angle was close to 90°, whereas the stabilization was ineffective with strongly polar oils due to the too little contact angle (Zhou et al., 2011). These results consisted with the condition that only solid particles with contact angle in a proper range can form Pickering emulsion. In order to solve the problem from previous studies, they subsequently modified Fe_3_O_4_ nanoparticles to increase their hydrophobicity, where the influence of coating type, extent, chain length and oil fraction on resulting Pickering emulsions were systematically discussed (Zhou et al., 2012).

**FIGURE 1:**
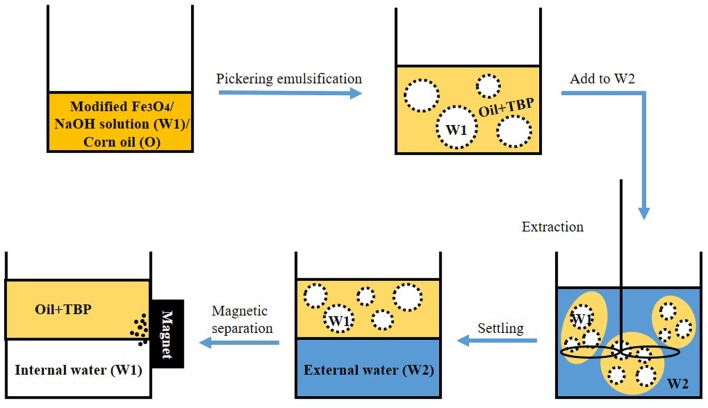
**Schematic illustration of the extraction process using magnetic particles stabilized Pickering emulsion (TBP is tri-*n*-butyl phosphate, used as the capture agent)**. Adapted with permission (Lin et al., 2016). Copyright 2016, Elsevier.

One of the most unique advantage of Pickering emulsions stabilized by magnetic particles is that they can be easily demulsified and reused by simply apply an external magnetic field. In this respect, one recent study set up a convenient system for the extraction of wastes from water. They employed hydrophobic oleic acid coated nano-Fe_3_O_4_ particles to firstly form W/O Pickering emulsion, and then W1/O/W2 came into being after adding the aqueous feed phase with organic waste (**Figure 1**) (Lin et al., 2016). This new type of extracting system will have extensive use in the future due to the convenient, recyclable, and reusable properties.

### Chitosan

Chitosan, the second abundant polymer in the world, is a linear polysaccharide produced by deacetylation of chitin (Wei et al., 2012b). The most outstanding and irreplaceable properties of CS are its biodegradability and biocompatibility due to the free amino and hydroxyl groups along its backbone, which makes it very useful in the field of biomedicine and pharmaceutics (Wei et al., 2012b). Moreover, CS is a particularly green polymer owing to its excellent solubility in dilute acid aqueous solutions.

**FIGURE 2:**
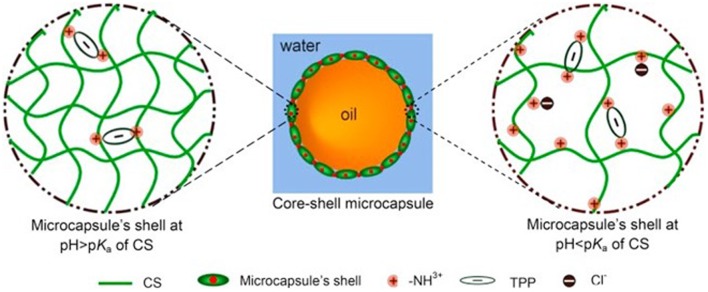
**An illustration of the influence of pH on the chitosan-tripolyphosphate shell structure**. Reproduced with permission (Mwangi et al., 2016). Copyright 2016, Elsevier.

In the research of Wei et al. (2012b), bare CS nanoparticles were used as the emulsifier to establish Pickering emulsion, and MCs were formed through evaporation of CH_2_Cl_2_. Besides, they also investigated the covalently cross-linked CS with glutaraldehyde. While in another work, a different cross-linking method was used, where CS particles were ionically cross-linked with sodium tripolyphosphate (TPP) after the formation of emulsion droplets, and the results indicated that the size of the MC products and the amount of oil leakage were affected by the pH used in the cross-linking process (Mwangi et al., 2016). Since at low pH, CS polymer chains got extended and the MCs became swollen, porous microstructures formed consequently and resulted in oil leakage (**Figure 2**) (Mwangi et al., 2016). In addition, a more detailed and systematic investigation of the CS-TPP nanoparticles-fabricated condition was carried out by another work, and the encapsulation of a bioactive compound curcumin as well as the release profile were also studied (Shah et al., 2016). Considering the physicochemical properties observed above, it is obvious that Pickering emulsions stabilized by CS can be controlled by regulating the processing conditions, like pH and CS particle-concentration, so that different applications and properties, such as delivery and stimulus reaction, can be achieved.

Apart from unlinked- or linked-CS particles, complex compounds consisting of CS and other molecules like surfactants or solid particles, were also utilized to form Pickering emulsions. For a more biocompatible purpose, a few studies have been conducted using CS-solid particles complexity as the emulsifier. One recent research fabricated Pickering emulsion stabilized by the complex of PS particles and CS, which appeared to be more stable than emulsions prepared from individual PS or CS particles (Zhang et al., 2015). They also proposed the mechanism that at lower CS concentration, the flocculation of PS particles was induced by the adsorption of CS, which served as the main emulsifier, whereas with the increase of CS concentration, the emulsion was stabilized by more free CS particles (Zhang et al., 2015).

### Cyclodextrin

Natural CD is a cyclic oligomer of α-D-glucopyranose, respectively, named α-, β-, and γ-CD. A CD molecule commonly has a shallow truncated cone shape with hydroxyl groups of glucoses facing the exterior end of the molecule, and a large hydrophobic cavity that can serve as the host of water-soluble molecules (Leclercq and Nardello-Rataj, 2016). Besides, being also biocompatible and non-toxic, CD is an attractive emulsion stabilizer as well, with potential usage in food, pharmaceutics, and skin care.

Previous studies have shown that CDs can form surface active complexes and microcrystals assembled at oil–water interfaces to stabilize emulsions (Li et al., 2014), which was proposed to start with molecular adsorption of CDs at the interface, and end up as stable Pickering emulsions due to the later-formed microcrystals (Wu et al., 2016). A more recent study gave a new approach to manufacture novel non-spherical Pickering emulsion droplets using a number of different kinds of oil, which illustrated that morphology and size of droplets were dependent on the type of oils and the concentration of CDs; besides, the result also confirmed increased stability and potential applications for drug delivery of non-spherical products (Wu et al., 2016).

**FIGURE 3:**
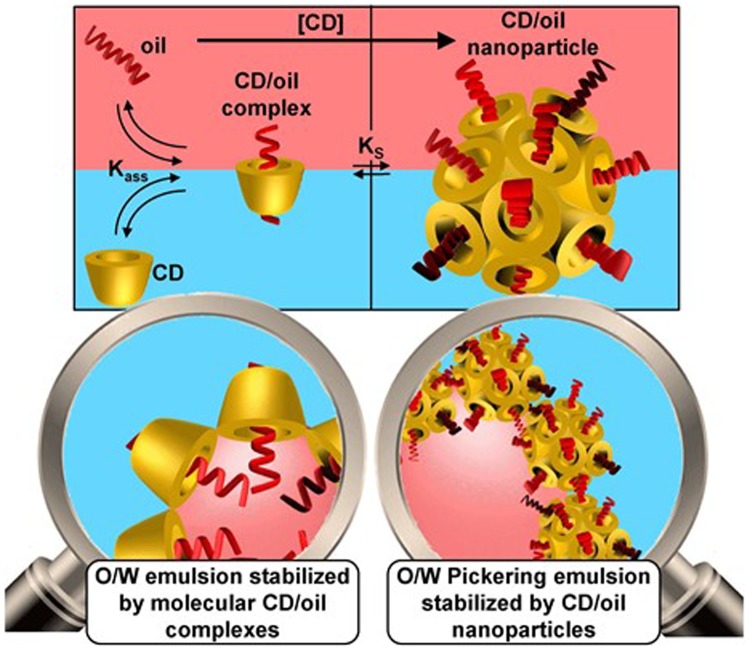
**Schematic illustration of the formation of O/W Pickering emulsion affected by the total CD concentration (*K*_ass_ and *K*_S_ are the binding and the solubility constants, respectively)**. Reproduced with permission (Leclercq and Nardello-Rataj, 2016). Copyright 2016, Elsevier.

In the research of Leclercq and Nardello-Rataj (2016), the mixture of oil, water, and native CDs resulted in the formation of very stable Pickering emulsions, of which the ability to stay against coalescence was proposed due to the formation of insoluble CD/oil inclusion complexes, and the complexes became nanoparticles when the concentration of CDs further increased (**Figure 3**). What’s more, this kind of emulsion could be used for sustainable delivery of antifungal econazole derivatives, and has been proved to be at least as active as commercially available ones (Leclercq and Nardello-Rataj, 2016), bringing about a promising method to fabricate useful antifungal systems, which will have potential medical value after systematic study in the future.

### Carbon Nanotube

Carbon nanotubes (CNTs) have appealed great interests in recent years due to their unique properties, like large surface area and more exposed active sites. Nevertheless, because of the hydrophobicity of CNTs, it is hard to disperse them well in aqueous solutions, thus most of former studies focused on preparation of W/O emulsions, whereas more useful researches about O/W emulsions were far less (Chen et al., 2011).

To increase the hydrophilicity of CNTs through an easier and higher-yielding method, several attempts have been done. One attractive approach was to treat CNTs with oxygen plasma to introduce hydrophilic functional groups, like hydroxyl and carboxyl groups, while no noticeable damage arose after the treatment; besides, it was notable in the experimental results that sonication time, CNTs concentration, and plasma treatment period had crucial influences on the size as well as size distribution of droplets (Chen et al., 2011). The same group also fabricated MCs by solvent evaporation of plasma-treated CNTs-stabilized O/W Pickering emulsion (Chen et al., 2010).

In addition, it has been proposed that compared to spherical emulsifiers, nanosheet-shaped particles can restrict the rotation of MSs to a larger extent at the water–oil interface, resulting in relatively more stable emulsions (Liang et al., 2011). For further applications, Shan et al. (2015) combined the catalytic potential of layered double hydroxides (LDHs) and the high thermodynamic stability of CNTs as emulsifiers to prepare Pickering emulsions, which could be effectively used as catalyst for the oxidation of alcohol (Shan et al., 2015). Their approach was fairly simple by just treating CNTs with acid and base sequentially for better dispersion, and growing LDHs on nanotubes after adsorption of metallic ion and nucleation.

### Natural Stabilizers

Some biological and food-grade particles have been increasingly employed in formulation of Pickering emulsions due to their excellent biocompatibility, biodegradability, as well as attractive potential applications in food and drug delivery fields. Fabrication methods and interfacial attachment efficiency tuning of these edible particles, research trend and challenges of employing them as Pickering emulsifiers have been well-reviewed by another article (Xiao et al., 2016). Among them, starch, zein, soy protein, whey protein, and bacteria-related particles served as Pickering emulsifiers will be talked over in this review.

#### Starch

Starch is a natural material that could be obtained from various botanic resources. Being biodegradable and non-toxic, starch granule is an excellent candidate to be applied in food industry, biomedicine, and so on. However, as a kind of native material from different resources, starch particles have broad size range, which leads to bad influence on its performance as an emulsifier. In addition, considering the poor hydrophobicity of starch granules, modification is necessary to more successfully stabilize O/W Pickering emulsions (Timgren et al., 2011; Tan et al., 2012).

One work from Li’s group compared four kinds of native starch granules from different resources in several aspects, such as particle size and configuration, surface charge, contact angle, emulsion stability, and surface morphology, in order to investigate factors that determined emulsion-stabilizing ability of starch granules (Li et al., 2013). It turns out that rice starch, which has the smallest particle size among the four, possesses the best capacity to form stable Pickering emulsion with the least amount of granules. This research came to the conclusion that it was the size of particles rather than morphology or surface chemistry that affected the ability of starch granules to stabilize Pickering emulsions. Besides, they also con-firmed that 80°C heating for 2 h changed Pickering emulsions to gel network. Moreover, another appealing study was carried out to dig out the influence of heat-treating on properties of starch-stabilized Pickering emulsions. Researchers found that proper heat treatment could make monolayer barrier formed by starch particles more dense due to the swelling of starch particles, thus providing products with more promising applications in drug-release and molecule protection (Sjoo et al., 2015).

It has already been verified that native starch granules could be tuned more hydrophobic after modification by octenyl succinic anhydride (OSA) (Rayner et al., 2014) while not affecting important characters of starch. Song et al. (2015) systematically looked into several major factors, including starch particle concentration, oil fraction, pH, NaCl concentration, and so on, that influenced stability of Pickering emulsions stabilized by OSA modified starch and optimized the parameters for soybean O/W Pickering emulsions.

#### Zein

Zein is an abundant material extracted from corn, which has been extensively studied in many fields (Shukla and Cheryan, 2001; Lawton, 2002). As a kind of water-insoluble food-grade protein, zein bears high proportion of hydrophobic amino acids, while the degree of hydrophobicity can be tuned by pH (Pomes, 1971).

It was proposed by de Folter et al. (2012) that zein colloidal particles could directly serve as Pickering emulsifiers without surface-modification, because the three phase contact angle θ was close to 90° when pH deviated from zein isoelectric point (Bornaee et al., 2014). Nevertheless, given that zein-stabilized emulsions were unstable when pH is close to the isoelectric point, and that pure zein particles couldn’t fully cover surface of droplets, modification has been done to form zein/NaCas nanocomplexes by adsorption of sodium caseinates (NaCas) onto zein nano-particles, which promoted the emulsive-ability of zein colloidal due to the amphiphilic of NaCas (Feng and Lee, 2016). Besides, through changing the zein:NaCas ratio, different surface cover results occurred.

#### Soy Protein

Soy protein is commercially available and non-toxic, even well nutritious, making it a promising food-grade material to establish Pickering emulsion systems (Miriani et al., 2012; Liu and Tang, 2013). Besides, their compatibility under high pressure-emulsifying condition results in much finer droplets (Liu and Tang, 2016a). In recent few years, great attention has been paid for the application of soy proteins like soy protein isolate (SPI) or its major component glycinin, especially the serial works done by the group of Tang (Luo et al., 2013; Liu and Tang, 2016a,b,c).

One of the studies mentioned above verified that a simple thermal treatment (at 95°C for 15 min) of SPI followed by addition of NaCl at an appropriate concentration could effectively form gel-like Pickering emulsions, with gel stiffness progressively increasing as the glycinin content raised (Luo et al., 2013). In addition, another study of them further revealed the importance of surface charge to the interfacial adsorption of pre-heated SPI, thus affecting emulsifying results (Liu and Tang, 2016b). The surface charge was tuned by different NaCl concentrations (0–500 mM), resulting in the conclusion that increase of ionic strength gave rise to an obvious decrease in drop size and surface coverage at the interface.

#### Whey Protein

Whey protein is a food-grade material produced during cheese-making process, which has been widely used in food industry. However, due to the need of heat treatment during food procedure, proteins tend to denature, resulting sequentially in instability of the emulsion (Dickinson and Parkinson, 2004). Despite the wide-using of whey proteins in other industries, it is a newly attempting method to apply them to preparation of Pickering emulsions as the emulsifier.

In order to solve the problem of instability after heating, heat-resistant whey protein isolate nanoparticles (WPI NPs) have been developed by Zhang and Zhong (2010) through heating WPI proteins within W/O emulsion droplets at 80°C for 15 min, where denatured proteins got cross-linked. Firstly utilizing WPI NPs as the emulsifier to form Pickering O/W emulsions, Wu et al. (2015) investigated some factors that influenced the steadiness of Pickering droplets, especially surface charge, where low charge resulting in weaker repulsions between particles as well as more hydrophobicity, thus causing instability of emulsions (Wu et al., 2015). Consisted with the relation between surface charge and emulsion stability shown above, another work using whey protein microgel (WPM) particles as the Pickering stabilizer also verified that charged particles could maintain proper distance between neighboring drops so as to prevent coalescence, whereas uncharged particles formed a continuous 2-D network because of aggregation (Destribats et al., 2014).

#### Bacteria-Related Particles

It has long been found that certain types of microorganisms could serve as emulsifiers (Srivastava et al., 1970), where surface properties of microbes, such as surface charge, functional groups, and special structures, are believed to play an important role (Firoozmand and Rousseau, 2016). Since some bacteria and yeast have already been extensively used in food, cosmetic, and medical industries, it is reasonable to believe that microbe-stabilized Pickering emulsions will have promising applications.

Considering negative charges existing on bacterial cell walls, and in order to improve the biocompatibility and emulsive ability of positively charged CS, Wongkongkatep et al. (2012) proposed a novel idea to establish a bacteria-chitosan network (BCN) through electrostatic interaction, which successfully promoted the formation of highly stable O/W Pickering emulsion. They have confirmed the applicability of this BCN to virtually any kind of organic solvents except ethyl acetate, and to any type of bacteria. Besides, the as-proposed method was very simple by direct self-assembly of the two ingredients on the oil–water interface.

More recently, Firoozmand and Rousseau (2016) gave a more detailed and systematic report about producing Pickering emulsions using yeast and two kinds of lactic acid bacteria as stabilizers. A worth-noting result was that these emulsions could be obtained at a high oil: water ratio (e.g., 4:1), when full droplet coverage and the close-packed oil droplet arrangement appeared to give gel-like emulsions (Firoozmand and Rousseau, 2016).

Studies pointing at using bacterial-related materials as Pickering emulsifiers are not as much as other solid particles such as silica, clay, or other food-grade stabilizers like whey proteins, partly because the practicability, safety, and large-scale productivity. More researches are needed in order to understand the mechanism more precisely and promote further applications to fully utilize this newly interested method.

## Classification of Products

Different raw materials, processing condition, and preparation methods give rise to different types of nano-materials, and there are many ways to give a classification. This review will roughly classify the nano-materials fabricated by Pickering emulsification as three parts: MS, MC, and JCPs, which are three mainly investigated types of Pickering emulsions and materials in recent years.

### Microsphere

Microspheres have raised great attention in the last three decades due to the demands of fine materials, mesoscopic science, nanotechnology, and so on (Kawaguchi, 2000). Bearing many attractive features, such as small size and volume, large specific surface area, good diffusion and dispersion ability, uniform or variation of size, special surface chemistry, novel morphology, and so on, MSs provide great facilities to different fields. Until now, numerous methods have been put forward to fabricate MSs, such as emulsion methods (single-emulsion process and double-emulsion process), phase separation, spray drying method, supercritical fluid method, and so on (Hu L. et al., 2013). Except above listed methods, Pickering emulsification is becoming an appealing choice due to many advantages like stability, feasibility, and uniform size of products.

As a representative of earlier studies about MSs, Fujii et al. (2009) described the first use of interaction between Hap nanoparticles and polymers to prepare Pickering emulsions, simply by mixing Hap water-suspension and PLLA dichloromethane solution and handshaking, followed by evaporation of oil (Fujii et al., 2009). As has been discussed in Section “Hydroxyapatite,” Hap can serve as Pickering emulsifier only when certain functional groups exist, hence in this example, they utilized the carbonyl groups carried along PLLA. On the other hand, by combining Hap and PLLA, they could realize both improved cell-adhesiveness and the drug-carrying ability provided by PLLA. Therefore, this report might be useful in drug delivery system, cell scaffold, and so on.

Besides, distinctive core-shell structure is widely studied among MSs, whereas the aim of well-defined structure and reproducible method through Pickering emulsification still has a long way to go. One outstanding work in this respect referred to monodispersed hydrolyzed poly(glycidyl methacrylate) (PGMA) MSs, which were prepared by a two-step dispersion polymerization, followed by hydrolysis, so as to transform the epoxy groups into glycol groups and to make PGMA slightly hydrophilic (Liu et al., 2015). It was confirmed that PGMA MSs without hydrolysis couldn’t stabilize Pickering emulsion, while the as-prepared hydrolyzed PGMA obtained the ability to do so, where they arrayed compactly on the surface of MSs, leading to the perfect spherical virus-like surface morphology.

In recent years, micro-scaffold is also a focus of research, due to the necessity of bone-tissue engineering. Regarding this, MS-incorporating scaffold has been extensively studied, whereas facile and low-cost preparing methods are still being explored. Hu et al. (2016) recently proposed a one-pot approach that applied Pickering emulsion template, involving *in situ* gelation of alginate (ALG) as well, to fabricate porous scaffold for dual-drug release and tissue engineering. An attracting part was that, in order to study the dual-drug release of their products, hydrophobic ibuprofen (IBU) was added to oil and hydrophilic bovine serum albumin (BSA) to water so that IBU was in MSs, and BSA was in scaffold matrix. According to their drug-release results, IBU hold a good release profile, while the cumulative release of BSA was faster, due to the two-stage release of inner-contained IBU and the difference in hydrophobicity of two drugs. In addition, concentration of Hap and D-gluconic acid δ-lactone (GDL) had significant influences on various properties of the scaffold, such as the swelling property and scaffold internal structures, which in turn would affect the application.

### Microcapsule

Microcapsules basically can be termed as MSs with one big cavity (Maeda et al., 2010), attracting much attention over the past decade due to their distinctive properties such as large surface area and surface permeability, which draw huge attention in areas of drug release, biomedicine, protecting sensitive molecules from environment, and so on (Chen et al., 2010). There have been various kinds of method regarding to the preparation of MCs, such as layer-by-layer (LbL) assembly, the use of microfluidic devices, solvent extraction, spray drying, via Pickering emulsification, and so on (Mwangi et al., 2016).

Melamine formaldehyde (MF) has been largely employed due to its mechanical and porous property. It was reported that pre-polymer of MF (pre-MF) could *in situ* polymerize after absorbing onto Hap-stabilized Pickering droplets to form a composite shell, and MCs came into being after solvent evaporation (Hu Y. et al., 2013). Besides, one layer of PMF could be another shell apart from solid particles, which reduces the porosity of products and promotes sustained drug-release behavior. Recently, Williams et al. (2015) utilized this principle to produce laponite/PMF double shell MCs with a cationic polyelectrolyte, Magnafloc, where the reaction of secondary amine groups on Magnafloc chains with MF facilitated deposition of MF.

Another important method to fabricate MCs is to make use of ionotropic gelation, where natural ionic biopolymers are able to form gel-like matrix by cross-linking with calcium ion. In order to obtain MCs with desired size and a large quantity, Leong et al. (2015) discarded nozzle and microfluidic devices, turning to combine ionotropic gelation and Pickering emulsion by letting biopolymer cross-link on the oil–water interface after CaCO_3_ nanoparticles stabilizing Pickering emulsions and adjusting pH. However, although different types of biopolymer and core-oil could be successfully applied into this novel method and produced products with good biocompatibility, this method required four steps, which would greatly reduce productivity and increase cost, facing the need of further optimization.

### Janus Colloidal Particles (JCPs)

**FIGURE 4:**
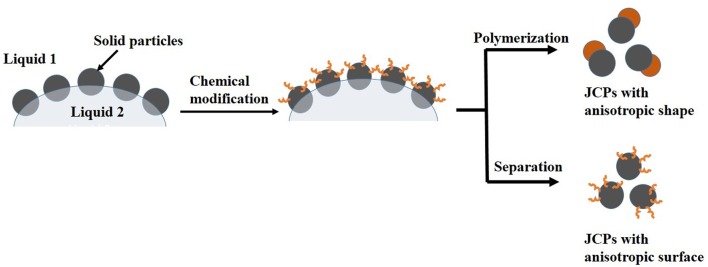
**Schematic diagram of two types of Janus colloidal particles**. Adapted with permission (Kaewsaneha et al., 2013). Copyright 2013, Elsevier.

Janus colloidal particles often bear two sides of different chemistry, like one side’s hydrophilicity and another side’s hydrophobicity, or possess novel non-spherical shapes. They have increasingly potential applications in material science, biomedicine, cosmetics, and so on. A great amount of works have come out relating to JCPs, and Pickering emulsion is considered as one of the most effective intermediates to prepare JCPs, due to its capability to control the geometry and functionality (Kaewsaneha et al., 2013). The basic process always involves chemical modification or etching of nanoparticles on oil–water interfaces of Pickering emulsions in one or two phases. Generally, JCPs can be divided into two categories: JCPs with anisotropic surface chemistry (Pardhy and Budhlall, 2010; Wang et al., 2014; Zahn and Kickelbick, 2014; Zenerino et al., 2015) and morphology (Gu et al., 2005; Liu B. et al., 2009) (**Figure 4**). With respect to JCPs, some representative works, preparation processes, as well as lucid analysis were presented in another review (Kaewsaneha et al., 2013).

With regard to Janus particles with two distinctive surface properties, Zahn and Kickelbick (2014) prepared amphiphilic titania nanoparticles at the oil–water interface in Pickering emulsion by adding hydrophobic coupling agents in oil phase and hydrophilic ones in water phase, both of which contain a phosphonate anchor group (Zahn and Kickelbick, 2014). Sequentially, they tested the performance of these amphiphilic titania nanoparticles as surfactants, which showed better performance than isotropic nanoparticles, due to better wettability of anisotropic particles both in water and in oil phase. This report clearly revealed that Pickering emulsion serves as a good template for the one-pot surface modification of nanoparticles, and that the anisotropic products are potential surfactants for emulsion formation.

As a classical and typical example of Janus particles with anisotropic shape, Liu B. et al. (2009) proposed a method to obtain tunable Janus particle shapes by asymmetric etching of nanoparticles at the Pickering oil–water interface, and it was a practical idea to employ solidified wax as the oil phase in order to prevent rotation of nanoparticles. Choosing silica colloids as the emulsifier could not only stabilize Pickering emulsions, but also conveniently be modified with silane interlayer and selectively etched in the aqueous phase to give non-spherical morphology. The experimental results indicated that the shape of Janus particles evolved from spherical to non-spherical with increased etching extent, obtaining mushroom-like, dimer-like and cap-like products. Besides, by drop-wise adding the silane monomer, the authors even fabricated nanoparticles with one side of nano-flowers.

Some complementary examples of micro- or nano-particles obtained by Pickering emulsion systems are summarized in **Table 1**.

**Table 1:** Some supplementary examples regarding to three types of materials originated from Pickering emulsions.

Type of products	Morphology and structure	Solid-particle stabilizers	Preparation procedure	Reference
1. Microspheres (MSs)				
HAp-coated biodegradable MSs	Spherical	HAp NPs	CH2Cl2(PLLA)/HAp/water-hand shaking-solvent evaporation	Fujii et al., 2009
PS/nano-SiO_2_ composite MSs	Spherical, core-shell structure	MPTMS modified SiO_2_ NPs	Styrene(AIBN)/nano-SiO_2_ composite/water-polymerization-drying	Zhang et al., 2009b
SiO_2_ NP-coated PLGA MSs	Spherical	SiO_2_ NPs	CH2Cl2(PLLA)/SiO_2_ NPs/water-hand shaking-solvent evaporation	Wei et al., 2012a
Core-shell polymeric MSs	Spherical virus-like, core-shell structure	Monodisperse P(GMA) MSs	Preparation of hydrolyzed P(GMA) MSs/P(GMA) MSs/water-hand shaking-polymerization	Liu et al., 2015
2. Microcapsules (MCs)				
CNT hollow MSs	Hollow spherical shell	O_2_ plasma-treated CNTs	CNTs-Plasma treatment-cyclohexane/CNTs/water-sonication-evaporation of solvent-washing, filtrating, drying	Chen et al., 2010
Nanocomposite polysaccharide MCs	Hollow spherical shell	Polysaccharide, alginate and chitosan	LbLA:Poly(ethylene imine) surface-modified laponite particles/O-LbL deposition of alginate and chitosan	Hao et al., 2013
Oil-core biopolymeric MCs	Spherical	CaCO_3_ NPs	Oil/CaCO_3_ NPs/water-emulsification-adding polyanionic biopolymer-CaCO_3_ NPs dissolution-ionotropic gelation	Leong et al., 2015
Ionically cross-linked CS MCs	Spherical	Ionically cross- linked CSPs	Oil/CSPs/water-reducing pH of the aqueous phase-the cross linking of CSPs	Mwangi et al., 2016
HAp/PMF-coated AAO-loaded MCs	Spherical	HAp NPs/PMF	AAO/HAp NPs/water-emulsification-polymerization of pre-MF-washing-drying	Hu Y. et al., 2013
3. Janus particles				
Laponite clay-P(divinylbenzene) with anisotropic surface potentials	Spherical	Laponite clay	P(divinylbenzene)/laponite nanoclay/water-wax/colloids/water-cation exchange	Pardhy and Budhlall, 2010
Janus Cu_2_(OH)_2_CO_3_/CuS MSs	Core-shell MS	Cu_2_(OH)_2_CO_3_ MS	Styrene/Cu_2_(OH)_2_CO_3_/water-sonication-polymerization-reaction with thioacetamide-dispersion in toluene and heating	He and Li, 2007
Fe_3_O_4_-Ag heterodimers	Spherical	Fe_3_O_4_	Oil/Fe_3_O_4_/Ag^++^ aqueous solution-ultrasonic emulsification-Oil/Fe_3_O_4_/Ag/water-functionalization	Gu et al., 2005
Hybrid silica/PS Janus colloids	Non-spherical	SiO_2_ NPs	Wax/SiO_2_/water-modification with silane-asymmetric etching	Liu B. et al., 2009

## Morphology

### Morphology of Solid Particles

Since solid particles are one most crucial factor influencing the stability of Pickering emulsions, morphology of these particles should be of great concern. Traditional and mostly used Pickering emulsifiers are spherical like, however, more other shapes, including flake (Kim et al., 2013; Lin et al., 2015; Erdenedelger et al., 2016), cylinder (He et al., 2007), ellipsoid (Madivala et al., 2009), wire (Patra et al., 2010; Yan et al., 2015), dumbbell (Tu et al., 2013) and so on, are in heated research in recent 20 years. Works that focused on the mechanism of stabilizing Pickering emulsions by non-spherical solid particles have been published, which demonstrated that this stabilizing effect not only relates to steric effect that has been discussed for spherical particles, but also may results from capillary forces on the oil–water interface (Bresme and Oettel, 2007; Madivala et al., 2009). Besides, a computational method was also created to analyze the interfacial activity of non-spherical particles, which at the same time demonstrated that the hydrophilic balance commonly used to estimate the effect of surfactants might have less effect in solid particle-stabilizing colloidal systems (Ballard and Bon, 2015).

The existence of shape-induced capillary attraction is clearly indicated in the experiments of Madivala et al. (2009), where Pickering emulsions were formed by the stabilization of ellipsoidal PS particles and hematite particles with different aspect ratio. It was confirmed that the stabilizing effect was dependent on aspect ratio of ellipsoid particles, which significantly affected the shape induced capillary attractions. Besides, the authors revealed that the contact line of particles and interfaces was not elliptical, but was deformed to an undulated saddle-like ellipse that strongly depended on both aspect ratio and contact angle (Madivala et al., 2009).

One example of nanowire as the Pickering emulsifier used silica nanowires to fabricate Pickering emulsions, of which the stability highly depended on the length of nanowires (Yan et al., 2015). Besides, whether W/O or O/W Pickering emulsions could be formed is related to the hydrophilicity of silica nanowires, as well as length of them.

**FIGURE 5:**
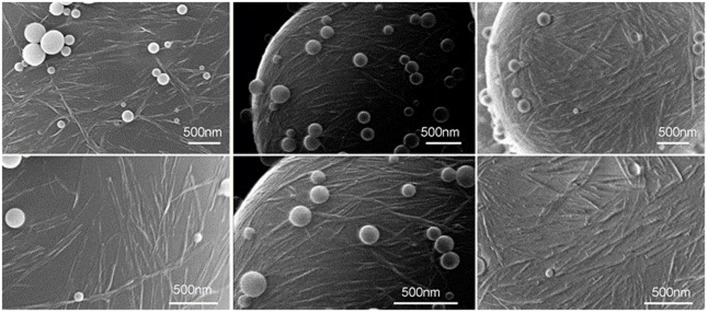
**SEM images for polymerized styrene/water emulsions stabilized by BCNs suspensions with different concentrations from left to right**. Reproduced with permission (Kalashnikova et al., 2011). Copyright 2011, Elsevier.

Bacterial cellulose nanocrystals (BCNs) with ribbonlike shape were used as a Pickering stabilizer to successfully generate hexadecane/water emulsions and styrene/water emulsions, where the polymerization of styrene carried out as the time of droplets forming. The coverage of droplets by cellulose nanocrystals can be clearly indicated in the characterization of styrene Pickering emulsion droplets stabilized by BCNS (**Figure 5**) (Kalashnikova et al., 2011). It was confirmed that surface coverage was constantly 60% when the concentration of BCNs was less than 5.2 mg/mL of hexadecane, while it reached 100% with BCNs concentration exceeding that boundary, which could even keep stable after centrifugation.

Flake-type nanoparticles are mostly from modified or unmodified clay and graphene particles. Kim et al. (2013) have synthesized polystyrene/laponite composite nanoparticles with flake-shaped modified-laponite as a stabilizer, which was confirmed by scanning electron microscopy and some other techniques. Besides, graphene and graphene oxide (GO) have been increasingly used as Pickering stabilizers for various applications such as rheological fluids, particles for supercapacitors, phase change materials, catalysis, and so on, which was well-reviewed by another work (Texter, 2015). Specifically, since GO is amphiphilic, it can be easily dispersed in solvents and induced onto the surface of droplets, thus performing as a good Pickering stabilizer. One the other hand, reduced GO (RGO), which can be produced by simply reducing GO, can also stabilize emulsions, especially for W/O type due to its less hydrophobicity. For example, thermally reduced graphene (TRG) was non-covalently modified with poly(vinyl alcohol) in order to increase the water-dispersibility, and then was utilized as an effective Pickering emulsifier to promote polymerization of methyl methacrylate (MMA) and encapsulate PMMA, the product of which had good conductivity and thermal-stability (Erdenedelger et al., 2016).

### Morphology of Products

Although hundreds of studies have been done to prepare useful nano-materials through Pickering emulsions, in most cases, the droplets of emulsions had a spherical geometry, which was resulted from the preference of minimizing surface energy (Aveyard et al., 2003). Nevertheless, contrary to the traditional thought that non-spherical droplets are unstable and of little use, recent studies have shown that emulsions with non-spherical drops could be as stable, and more importantly, non-spherical materials are even more ascendant than spherical ones in various fields such as drug loading and release, circulation, targeting, cellular uptake and so on, due to the different traction on different sides and surface characters (Simone et al., 2008).

Considering that oil and emulsifying solid particles are two most important components in a Pickering emulsion, the content and types of them are significant to the morphology of droplets. In this respect and for the first time, Wu et al. (2016) prepared non-spherical Pickering emulsion droplets (NSPEDs) with different shapes, such as discal, oval and rod-like shape, using CDs as the emulsifier, which was due to the various amount and distribution of the self-assembled CD microcrystals. They confirmed the proposed mechanism of the non-spherical droplets formation by tuning the oil: CDs ratio and then diluting the emulsion, when they observed changing from non-spherical to spherical droplet shape. Besides, Liu et al. (2011) fabricated Hap/poly(L-lactide-co-ε-caprolactone) composite micro-particles with morphology of both sphere and plate.

**FIGURE 6:**
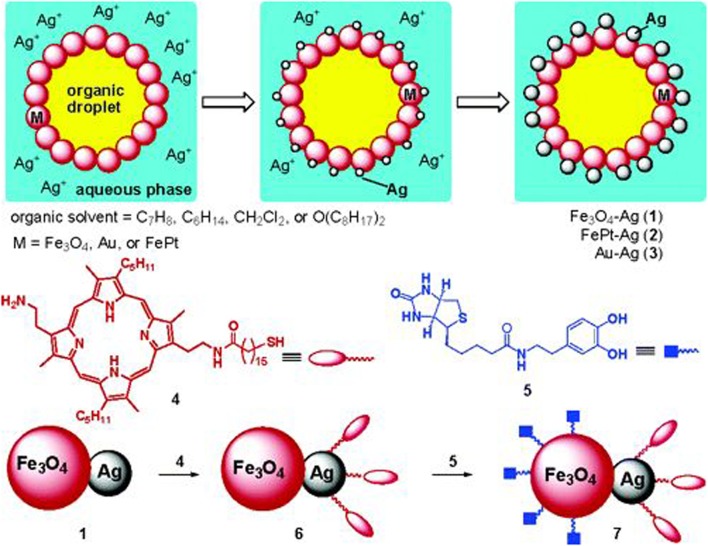
**The schematic refers to nanoparticles self-assembling at O/W interface firstly, and then a heterogeneous reaction taking place on the surface of the nanoparticles exposed to water phase to produce the heterodimers with two distinct nano-spheres**. Reproduced with permission (Gu et al., 2005). Copyright 2005, American Chemical Society.

In addition, preparing Janus colloids through Pickering emulsions is a promising method to produce non-spherical Pickering droplets. As has been mentioned in the former part, Janus particles with tunable shapes have been prepared by asymmetric etching of the colloids fixed at the oil–water interface (Liu B. et al., 2009). Besides, a more previous example was to form heterodimeric nanostructures, in which after Fe_3_O_4_, Au, or FePt nanoparticles self-assembling at the oil–water interface, a heterogeneous reaction took place at the surface exposed to water phase because of the partly catalytic effect of Fe(II) for the seeding of Ag nanoparticles (**Figure 6**) (Gu et al., 2005).

Furthermore, bearing a “standing” like appearance, the gel network formation of Pickering emulsions could render emulsions some unique and beneficial characters, like better drug-release profile and enhanced stability. This gel-like emulsions have been prepared mostly by food-grade proteins serving as Pickering emulsifiers, like pea protein (Shao and Tang, 2016), soy glycinin (Luo et al., 2013; Liu and Tang, 2016c), starch particles (Rayner et al., 2012), and so on. The group of Tang has done a series of works with regard to gel-like emulsions. One of them proposed origins of the formation of gel-like emulsions, including oil fraction, protein concentration, and the mode of emulsification (Luo et al., 2013).

## Biomedical Applications

### Drug Delivery and Release

Pickering emulsions can be stabilized by solid particles that are more biocompatible, such as CSs, CDs, and food-grade materials, as have been discussed in Section “Solid Particles.” Thus, products from these emulsions tend to be more biodegradable and properly used *in vivo*. In addition, a dense shell of solid particles will form around Pickering emulsion droplets acting as a barrier, and in some cases, internal polymers are able to interact with loaded-drugs, so sustained drug-release profile can be better achieved (Frelichowska et al., 2009). An effective treatment not only needs effective drugs, but equally importantly, needs proper drug-delivery and release vehicles to specific sites. For all the reasons above, we can come to the conclusion that Pickering emulsion is a prospective vehicle for controlled delivery, and it has indeed been deeply investigated in recent years (Marto et al., 2015).

Apart from features stated above, other special characters of obtained materials, like magnetic property derived from magnetic nanoparticles, can render products better usefulness as drug-vehicles. For example, polymer/SiO_2_ double-shelled capsules with hydrophobic magnetic Fe_3_O_4_ nanoparticles wrapped in polymer inner shell were prepared (Zhang et al., 2009a). It was confirmed that Fe_3_O_4_ nanoparticles in the products could be collected by external magnet, and disperse again after removing the magnetism, with no negative influence to the products. Thus, the products could be effectively directed to targets, collected and reused without aggregation. Besides, IBU was chosen as a model drug in this study, which exhibited a sustained release behavior because of the more impermeable double shell.

Regarding to the impending requirement of biodegradable drug-release vehicles, poly(lactic-co-glycolic acid) (PLGA) is a promising reagent due to its ascendant biodegradability with no need of removal. In this respect, pure PLGA micro-particles could be an ideal goal for *in vivo* drug delivery, which has already been fabricated by firstly forming Pickering emulsion using SiO_2_ nanoparticles as stabilizer, and etching off SiO_2_ after solvent evaporation (Wei et al., 2012a). Drug-loading and drug-releasing experiments indicated that pH had significant influence on both IBU-loading efficiency and releasing profile. As a whole, due to the biodegradability and biocompatibility of PLGA, as well as its impediment effect to burst release of drug, it has a broad developing prospect for drug-delivery.

In addition, as has been referred to, gel-like emulsions could further slowdown the release of drugs than common emulsions, and it seemed from experiments that the release profile was related to the stiffness of gel network (Liu and Tang, 2016c), which was affected by oil fraction and protein concentration (Luo et al., 2013).

### Porous Scaffold

Porous biomaterials serving as tissue scaffolds is catching considerable attention due to possible applications in tissue engineering (Peter et al., 2010a,b), since these scaffolds can provide biomimetic cellular environment for cell proliferation and differentiation, as well as physical support for newly formed tissues (Liu X. et al., 2009; Chen et al., 2012). Whereas ideal biologic scaffolds for bone tissue engineering should be poly-porous and properly interconnected, with enough mechanical stability, high bioactivity and biodegradability, as well as enough protein adhesion and adsorption ability (Hu et al., 2014). Various methods have been applied to prepare porous scaffold materials, while Pickering emulsion as template is a most prospective candidate due to its stable, facile processing and shorter time-consuming properties. Besides, choosing solid particles and inner materials with good biodegradable properties, it is safer for *in vivo* usage.

**FIGURE 7:**
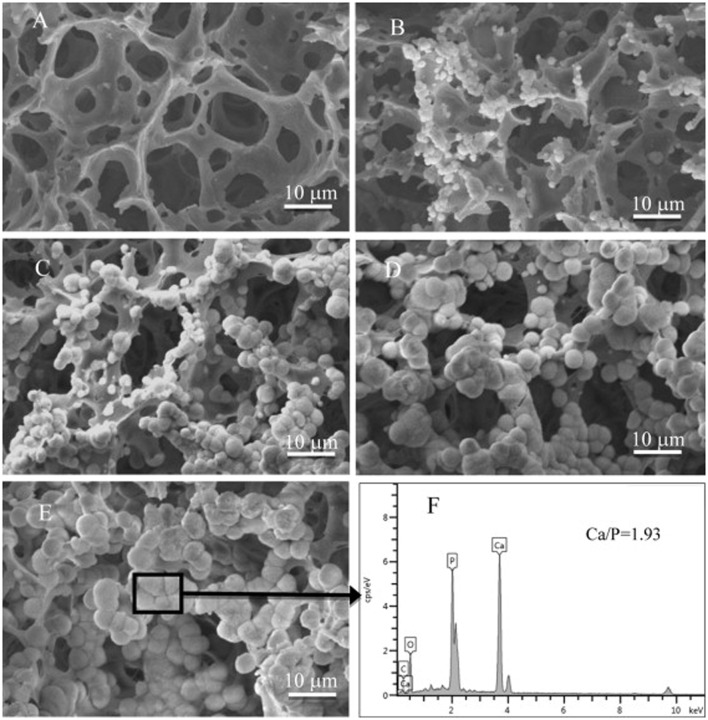
**SEM images of the Hap/PLLA NC scaffolds before (A)** and after mineralization for 7 **(B)**, 14 **(C)**, 21 **(D)**, and 28 **(E)** days; X-ray energy dispersive spectrometer (EDS) spectrum **(F)** shows the mineral composition on the area in **(E)**. Reproduced with permission (Hu et al., 2014). Copyright 2014, Elsevier.

Because of the good cyto-compatibility, protein adhesion ability, facile production, and strong mechanical property, Hap is a most commonly used Pickering stabilizer for biologic scaffolds. Besides, PLLA is also largely chosen as the internal material for Pickering emulsion because of its biodegradability. Thus, Hap/PLLA nanocomposite (NC) scaffolds were fabricated by solvent evaporation from Pickering emulsions, giving rise to highly porous structures usable for tissue engineering (Hu et al., 2014). *In vitro* mineralization experiments of as-prepared NC scaffolds in a simulated body fluid were carried out, showing good apatite mineralizing performance (**Figure 7**). More recently, the same group made use of *in situ* gelation of ALG to fabricate PLLA MS-incorporated porous scaffolds (Hu et al., 2016). Specifically, the process of ALG *in situ* gelation utilized the principle that, when D-gluconic acid δ-lactone (GDL) hydrolyzed in aqueous phase, Ca^2+^ released from Hap due to decrease of pH, as a result of which ALG cross-linked with Ca^2+^ to form stable ALG-Ca gelation. As well, *in vitro* cell culture experiments verified that mouse bone mesenchymal stem cells could proliferate on the scaffolds in a good manner. Both of these two studies took advantage of strength of Hap as a filler inside the matrix and its adhesion ability so as to overcome the disadvantages of PLLA. However, basic objectives of these two works were different. While Hap/PLLA NC scaffolds were designed to make PLLA as matrix after solvent evaporation, the newer ones let ALG-Ca gelation to be matrix, with the aim of incorporating PLLA MSs as the second medium for the build of dual-drug delivery system.

In addition, given that degradation rate of PLLA as scaffold materials is relatively long, and that degradation period can be tuned by polymer composition and molecular weight, an earlier study has employed poly(L-lactide-co-ε-caprolactone) copolymer to improve the biodegradability of scaffolds (Liu et al., 2011).

### Environment-Responsive Material

Smart stimuli/environment-responsive materials, whose properties can be altered dramatically by external stimuli, such as temperature, pH, ionic strength, electric/magnetic fields, or light, have attracted much attention because of their potential applications in medical and biological fields, such as controlled drug release, protein separation, and so on (Zhang et al., 2010). Pickering emulsification is one well-developed method that could incorporate raw materials with stimulating respondents to produce smart products.

#### Thermo-Responsive Material

Poly (*N*-isopropylacrylamide) (PNIPAM) is a most widely employed material for the fabrication of thermo-responsive products due to its lower critical solution temperature (LCST) in aqueous solution at 32°C (Gao et al., 2009). LCST refers to the critical temperature below which the components of a mixture are miscible for all compositions, and the LCST of PNIPAM was proposed to originate from its linear chain, which presented a coil-to-globule transition as the temperature was raised above the LCST (Zhang et al., 2010). Besides, the fact that LCST of PNIPAM is close to physiological temperature (37°C) makes PNIPAM have extremely promising applications in *in vivo* controlled drug release (Zoppe et al., 2012).

Gao et al. (2009) utilized PNIPAM to fabricate thermo-sensitive hybrid MCs, the result of which interestingly indicated that, consisting with the prediction from LCST, hollow MCs with SiO_2_/PNIPAM composite shells could be obtained when the reaction temperature was above LCST of PNIPAM, whereas core-shell MCs with SiO_2_ shell and PNIPAM-gel core were produced at the temperature lower than LCST. These changes of products could be clearly revealed by microscopic photos, fluorescence images, and SEM images.

In another study, Zhang et al. (2010) prepared capsules with hybrid shells, one component of which was PNIPAM, from an inverse Pickering emulsion. They carried out drug-release experiments, confirming that drug release rate of IBU increased with the increase of temperature at a proper range, which was due to the shrink of PNIPAN-inner shell causing from the loss of hydrogen bonds, thus squeezing drugs inside it out faster. This novel property could be effectively used to control drug-release profile by simply tuning the temperature.

#### pH-Responsive Material

Generally speaking, pH affects the stability of Pickering emulsions through modifying surface charge of solid emulsifiers (Wang et al., 2014) and thus changing electrostatic interactions between particles or molecules. Therefore, using pH-sensitive solid particles as the emulsifier can render Pickering emulsions pH-responsive property.

By copolymerizing methacrylate sulfadiazine with NIPAM, the copolymer stabilized Pickering emulsion not only obtain temperature-sensitivity from PNIPAM, but also got pH-dependence because of the amount of sulfonamide groups on methacrylate sulfadiazine (also affected by NaCl concentration) available for ionic interaction with doxorubicin (DOX) molecules, which in turn influenced the drug-release property (Zhou G. et al., 2013).

Another work utilized palygorskite particle, a kind of natural clay mineral with a lot of Si-O, Al-O, and Mg-O groups on the particle surface, to stabilize Pickering emulsions, which turned out to show *in situ* emulsification-demulsification cycles by adding HCl or NaOH (Lu et al., 2014). The pH responsive ability of as-prepared emulsions was due to the deprotonation and protonation of O-H groups on palygorskite particles, and consequently influenced the repulsion between solid particles as well as their emulsive ability and the stability against aggregation of oil droplets.

#### Other Stimuli-Responsive Material

Electric- and magnetic-responsive emulsions are another two types of intelligent functional emulsion, whose mechanical property and many other characters respond controllably to external electric and magnetic fields, respectively. One recent review discussed in detail about Pickering emulsion polymerization with GO, clay, and SiO_2_ stabilizers for electrorheological (ER) materials, together with Fe_2_O_3_ and Fe_3_O_4_ particles for magnetorheological (MR) materials, as well as their electric/magnetic-responsive behaviors (Piao et al., 2015). Among many studies referring to this respect, a work loading iron oxide nanoparticles onto RGO formed magnetically responsive Pickering emulsion, of which droplets could move by attraction of a permanent magnet without damage or contortion (Lin et al., 2015). This motion was confirmed to be related to the size of droplets, and will have promising usage for drug-transportation to certain locations by an external magnetic field. Specifically, since weaker magnetic fields should be employed for *in vivo* treatment, Pickering emulsions with relatively bigger droplets should be used so as to achieve quicker motion, according to this study.

Besides, Gu et al. (2015) based on the fact that, Se–Se bond would be very active and sensitive to dual redox environment, to prepare alginate microgels (Algms) with amphiphilic diselenide polymer aggregates as the Pickering emulsifier. They proved that drug delivery controlled by breaking off Se–Se bonds in either oxidative or reductive environment were successfully achieved. Development of other redox environmental-responsive materials like this may have great potential in target drug release.

## Other Applications

### Catalytic Facilitation

Apart from biomedical applications, nano- or micro-materials obtained from Pickering emulsions have also attracted increasing interests in fine chemistry, such as catalysis, due to their larger interfacial areas, which could largely improve catalytic efficiency. Besides, some novel properties of Pickering emulsions can also bring other advantages to catalysts compared with traditional emulsions, such as easier separation and recovery after reaction, as well as selective catalysis, which will be discussed in Section “Catalysts’ Separation and Extraction” and “Selective Recognization” (Huang and Yang, 2015).

As has been mentioned in Section “Cyclodextrin,” CD is able to include guest molecules, the complex of which can serve as a Pickering emulsifier. While guest molecules may just help promote the performance of stabilizers, they can also be a substrate of an oxidation reaction. For example, in the work of Leclercq et al. (2013), it was validated that oxidation of liquid olefins, organosulfurs, and alcohols could be effectively carried on in an O/W Pickering system, where substrate molecules formed inclusion complexes with CD molecules and acted as Pickering emulsifiers, with the addition of H_2_O_2_ as the oxidant and Na_3_[PW_12_O_40_] as the catalyst. Since the oil phase in this system is also the oxidized substrate, it can be regarded as a solvent-free system, which not only cuts down cost, but also produces less pollutant.

Photocatalysis is one mostly used approach for the purpose of organic contaminant degradation, while the efficiency of catalysts will reduce as the decrease of contaminant concentration. Thus, breakthroughs in this field will come into being when contacting area of contaminants and photocatalysts is enlarged. In the last two decades, Pickering emulsion has been explored as one of the methods to increase the efficiency of organic pollutants degradation, where nanoparticles like ZnO or TiO_2_ acted as emulsifiers as well as photocatalysts. In two representative studies (Wu et al., 2010; Nsib et al., 2013), inorganic nanoparticles were modified with organic molecules, such as titania, silane, or salicylic acid (SA), in order that the wettability of modified nanoparticles was suitable to serve as Pickering stabilizers. According to the research of Nsib et al. (2013), modifications with aromatic rings could increase the adsorption of phenyl molecules due to phenyl group interaction, and on the other hand, enlarge the response range of wavelength. Besides, they confirmed the increasing catalytic efficiency of photocatalysts after forming Pickering emulsions, by comparing with a mixing solution without emulsification.

In addition, the well-known advantages of porous materials are their larger total surface areas and porous structures, which provide more active reaction sites and diffusion channels. In this respect, mesoporous modified-silica nanoparticles were employed as both Pickering emulsifiers and catalytic reaction sites (Zhao et al., 2016). By comparing mesoporous silica with non-porous silica that had similar size, it was found that catalytic efficiency of Rh-contained catalyst in the former case was much higher than in the latter case, revealing the significant influence of porous structure on catalytic efficiency. To be specific, silicas that have porous structure provided not only a more uniform distribution for catalysts, but also abundant pores for reactions as well as diffusion of products and reagents, while reactions could only take place at the interface in the case of non-porous silica nanoparticles.

Moreover, mesoporous organosilica could effectively enhance biocatalytic performance of lipase by immobilizing it, which reveals promisingly potential use in biodiesel production (Jiang et al., 2014). Underlying reasons for this immobilization include promotion of protein refolding in the mesoporous channels, facilitation of lipase loading capacities due to functional groups that regularly distribute within the framework, as well as prevention of lipase leakage (Wang et al., 2010).

### Catalysts’ Separation and Extraction

Not only are Pickering emulsions proved to be attractive templates for efficient catalysis, but they may provide a feasible way of separating and thus extracting solid catalysts as well. In this way, the goals of low-cost, green-production, less catalysts-loss, shorter time-consuming can be achieved, which promotes industrial applications of these proposed plans.

A convenient way to extract products involves magnetic particles as solid emulsifier, which could be demulsified using a magnet (Lin et al., 2016). However, destroying obtained emulsions is time- and energy-consuming, thus urging for better solutions. One promising method is to collect products by simple phase-inversion of Pickering emulsions from O/W type to W/O type and directly separating the product-containing oil phase, without the need of demulsification and filtration. This goal could be realized by adding acid or base to tune the hydrophilicity of solid particles locating at oil–water interface, and in turn bringing about inversion of Pickering emulsions, which was confirmed to be able to recycle for at least 36 times (Yang et al., 2013; Yu et al., 2014).

**FIGURE 8:**
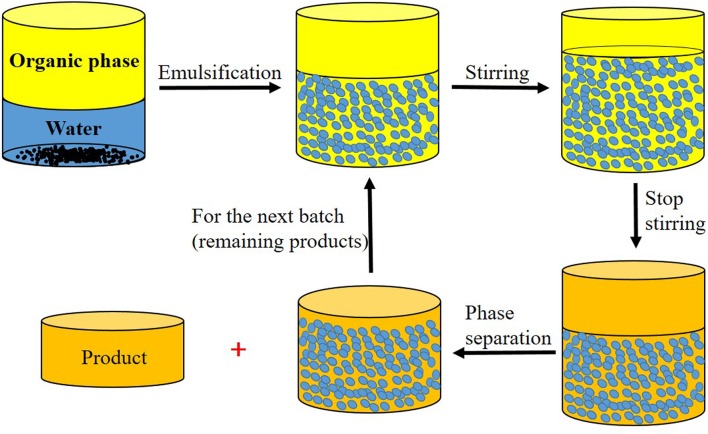
**Schematic illustration of the proposed PEOBS for catalysts separation and recycling**. Adapted with permission (Liu et al., 2014). Copyright 2014, WILEY-VCH.

Since the separating and recycling method mentioned above needed to inverse the emulsions, it still consumed certain time and energy. Another novel while more handy approach put forward by the same group is to increase the oil fraction and produce Pickering emulsion/oil biphasic system (PEOBS), making use of the gravity force after ending stirring process, where most of products remained in oil phase owing to smaller fraction of oil in Pickering emulsions (**Figure 8**) (Liu et al., 2014). They have verified the recycling performance of pH sensitively modified silica, TiO_2_, as well as mesoporous SiO_2_ which acted as emulsifiers, and all got good results, showing general practicability of this method.

### Selective Recognization

It is an important focus to detect and separate some toxic and contaminative molecules from body or environment efficiently and selectively. Molecular imprinting technique (MIT) and its products, molecularly imprinted polymers (MIPs), have been considered a most efficacious technique to selectively recognize and remove target molecules. MIPs can be prepared through Pickering emulsions by firstly polymerizing monomers in the oil phase of Pickering emulsions, and then removing template molecules under appropriate conditions, which will produce cavities inside the material with specific size, shape, and 3-D structures suitable for selectively recognizing target molecules (Ou et al., 2015).

Considering that some samples are complex and difficult to separate directly by methods like chromatography, other solid-phase extraction (SPE) approaches should be developed as pre-treatment. In one work from Yang et al. (2015), MIP microsphere (MIPMS) were fabricated to extract bisphenols (BPs), which have certain toxicity to human body, from human urine samples (Yang et al., 2015). Adsorption experiments verified a more effective result of as-prepared MIPMS than non-imprinted polymer microsphere (NIPMS) for selective extraction of eight kinds of BP from samples. Thus, it predicted a promising method of combining with HPLC for detection of BPs, as well as other likely substances, more efficiently and selectively. Besides, the study of Ou et al. (2015) confirmed that adsorption capacity of template molecules was lower than that of structural analog molecules in MIPs and MNIPs (molecularly imprinted polymers), which meant that MIPs are likely to have important functions in distinguishing structural analogs.

A natural problem arouse about how to separate MIPs after extraction for reutilization, and magnetic particles again were chosen as a solution (Ou et al., 2015). For example, Zhu et al. (2014) synthesized magnetic molecularly imprinted microspheres (MMIMSs) employing yeast particles as stabilizers, and the products were used for selective adsorption of a contaminant, aaa-cyhalothrin (CL), from aqueous solutions (Zhu et al., 2014). Experimental results indicated that their Fe_3_O_4_-embeded products could be successfully separated and reused for at least three cycles, providing a practical prospect of magnetic carriers-contained recognizing products.

## Conclusion

With the increasingly large demand in food, cosmetic, pharmaceutical, tissue engineering field, Pickering emulsion has become a research hotspot because of the preparing simplicity, high stability, unified size range of products, and biocompatibility. Stabilized solely by solid particles, Pickering emulsions are stable against coagulation mainly due to the mechanical barrier that solid particles form at the oil/water interface. Besides, abundant characters, morphology and applications of materials fabricated from Pickering emulsion method are also attributed to diversified solid particles, which bear tunable properties, different wettability, helpful functions, and various morphology.

In this review, different aspects of Pickering emulsions are discussed in detail. Firstly, a great number of practical solid particles used for fabrication of Pickering emulsions are described, including some specific examples as well. Secondly, a classification of materials prepared through Pickering emulsion method to three main types are discussed, consisting of MSs, MCs, and Janus particles. Then, different kinds of solid particle-shape and droplet-morphology in Pickering emulsions, apart from sphere, are investigated. Finally, a large amount of applications of materials produced from Pickering emulsion systems, including both biomedical and other chemical or physical applications, are talked over.

However, even though many efforts have been done for the development of Pickering emulsion, some aspects still need further advancement. For one thing, theories that can fully and precisely explain the mechanism of Pickering emulsification are still in demand, especially for cases involving non-spherical solid particles as stabilizers. For another, applications of Pickering emulsions in biomedical field require more clinical experiments in order for real benefits to treatment of diseases. Thus, we expect more practicable usages of Pickering emulsification after future research attentions.

## Author Contributions

YY, ZF, XC, WZ, YX, YC, ZL, and WY participated in its design, searched databases, extracted and assessed studies and helped to draft the manuscript. WY conceived the initial idea and the conceptualization, participated in the data extraction and analysis, and revised the manuscript. All authors read and approved the final manuscript.

## Conflict of Interest Statement

The authors declare that the research was conducted in the absence of any commercial or financial relationships that could be construed as a potential conflict of interest.
